# Microbial co-habitation and lateral gene transfer: what transposases can tell us

**DOI:** 10.1186/gb-2009-10-4-r45

**Published:** 2009-04-24

**Authors:** Sean D Hooper, Konstantinos Mavromatis, Nikos C Kyrpides

**Affiliations:** 1 Department of Energy Joint Genome Institute (DOE-JGI), Genome Biology Program, Mitchell Drive, Walnut Creek, CA 94598, USA

## Abstract

**Background:**

Determining the habitat range for various microbes is not a simple, straightforward matter, as habitats interlace, microbes move between habitats, and microbial communities change over time. In this study, we explore an approach using the history of lateral gene transfer recorded in microbial genomes to begin to answer two key questions: where have you been and who have you been with?

**Results:**

All currently sequenced microbial genomes were surveyed to identify pairs of taxa that share a transposase that is likely to have been acquired through lateral gene transfer. A microbial interaction network including almost 800 organisms was then derived from these connections. Although the majority of the connections are between closely related organisms with the same or overlapping habitat assignments, numerous examples were found of cross-habitat and cross-phylum connections.

**Conclusions:**

We present a large-scale study of the distributions of transposases across phylogeny and habitat, and find a significant correlation between habitat and transposase connections. We observed cases where phylogenetic boundaries are traversed, especially when organisms share habitats; this suggests that the potential exists for genetic material to move laterally between diverse groups via bridging connections. The results presented here also suggest that the complex dynamics of microbial ecology may be traceable in the microbial genomes.

## Abstract 2

Interactions between microbial communities are revealed using a network of lateral gene transfer events.

## Background

Microbes dominate the planet, inhabiting a wide range of environments, including many previously thought to be too extreme or inhospitable for life. Identifying the habitat(s) occupied by a particular microbial organism is not a straightforward task. Often the initial habitat assignment stems from where the organism was first isolated, which may not be its only, or even its preferred, habitat. This is an increasingly frequent occurrence as more microbial species are being identified from metagenomic samples such as soil [1]. Furthermore, given the anthropocentric perspective of microbiology, it is not surprising that many bacteria have been associated with their location in the human body, even if this pathogenic phase constitutes only one part of their life cycle. For example, highly versatile, opportunistic pathogens in the *Pseudomonas *family are found in a wide range of habitats (for example, [2]), not only in humans or other hosts. Add to this the wide dispersal of microbes by physical processes [3,4] and the variation over time of the microbial community at one location, and the task becomes ever more complex.

Here we explore a new approach to study the interaction between microbes in various habitats based on the cohabitation history recorded in each microbe's genome. Rarely is one species found alone in its local environment. Even in highly specialized niches, such as acid mine drainage, the biofilms present are populated by more than one species [5]. Studies of other environments such as farm soil [1] and the termite gut [6] suggest a diversity that is difficult to capture even with large-scale metagenomic sequencing projects. This diversity creates opportunities for an organism to interact with a multitude of closely or distantly related neighbors in numerous ways, including possible lateral gene transfer (LGT) [7].

Since we cannot directly observe these interactions, we must use sequence data as proxy. For this purpose, we chose transposases that transfer both within and between genomes via paired insertion sequences (ISs) [8-10]. Transposases are potentially transferred laterally more frequently than many other genes, based on the low levels of divergence [11] compared to other genes. A further advantage of transposases over other protein-coding genes is lower degree of selection effects such as conservation, recombination [12], adaptive radiation [13] or counter-selection [14]. All of these issues make it difficult to track their movement between species and to determine whether they are laterally transferred or not. Transposase sequences, on the other hand, are under selective pressure to retain their ability to move between organisms, and tend to be removed from the genome if this ability is lost. Thus, they are well suited to provide a recent historical record of LGT events between microbes due to their mobility.

We analyzed the distribution of transposases among all sequenced microbial genomes, focusing on shared transposases that were most likely acquired by LGT. From these connections, we constructed a microbial interaction network including nearly 800 organisms. Since LGT between two taxa implies a shared habitat at the time of transfer, or alternatively the presence of a vector of transmission, these connections provide a means for evaluating current habitat assignments. Furthermore, connections between distant taxa were of particular interest, as they imply that the obstacles limiting transfer of genetic material across large phylogenetic distances can be overcome.

## Results

### Illustration of concept

**Figure 1:**
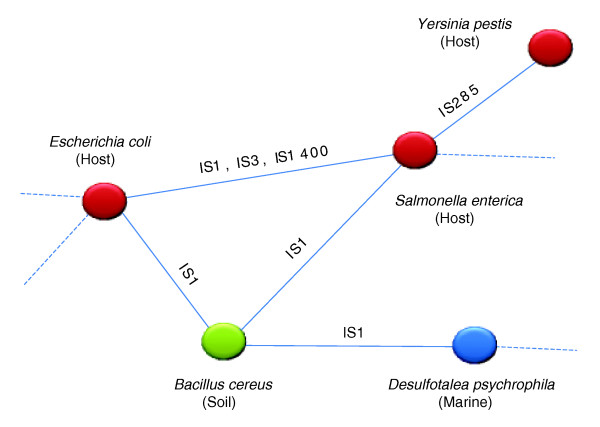
A conceptual representation of the transposase connection network. Nodes represent taxa, and edges signify the presence of one or more shared transposases. The transposase family is marked along the edge. Taxa are also colored depending on their habitat annotations.

**Figure 2:**
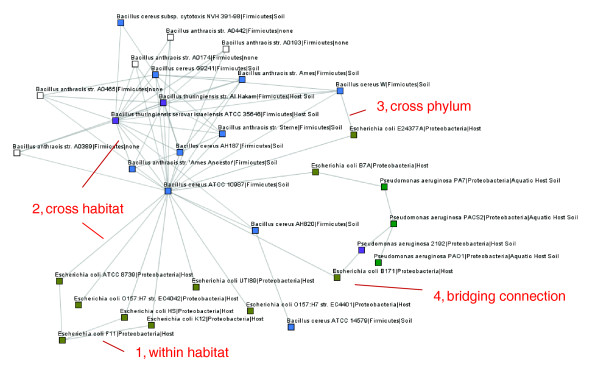
A subset of the full network illustrating concepts such as within-habitat connections (item 1), connections between habitats (item 2), connections that traverse phyla (item 3), and taxa that form bridging connections between other taxa that lack direct connections (item 4). Nodes are annotated by their species name, phylum and habitat annotation.

The complexities of transposase connections between taxa are best visualized as a network. Figure 1 illustrates the basic concepts of how this network was created. First, we represent each taxa as a node (circles) in the network. Second, we color the nodes corresponding to the habitat annotation. Finally, we search for any shared transposases between taxa. In Figure 1, for instance, we see that *Escherichia coli *and *Salmonella enterica *share members of three transposase families; IS1, IS3 and IS1400. We then connect these nodes by a single edge, representing the shared transposases. In this fashion, we gradually build the network and connect additional taxa. Each of the steps involved in forming the network is detailed below. Ultimately, the result is a large network of 774 taxa from 13 bacterial, eukaryotic, and archaeal phyla (Table S1 in Additional data file 1), connected by one or more transposase families. Figure 2 is a collection of representatives of three groups of organisms that have been extensively studied in microbiology; the *E. coli *group, *Pseudomonas aeruginosa *and various *Bacillus *strains. This figure is a subset of the full network for the purpose of illustrating specific concepts in this work.

### Transposase genomic context and vertical inheritance

A transposase may be shared between two taxa as the result of two distinct mechanisms: LGT and vertical inheritance. Sometimes, both mechanisms are involved, as when recently diverged species retain transposases that had been acquired by a common ancestor through LGT. In this study, we focus on transposase co-occurrences that most likely arose through recent acquisition by LGT. In order to distinguish between co-occurrences resulting from these two processes, we compared the genomic regions adjacent to the transposases within both taxa. Conservation of those regions would be a clear indication that these transposases were inherited from a common ancestor.

Transposases located within the same gene neighborhood (see Materials and methods) accounted for 5,641 co-occurrences between 685 taxa, while those residing in differing neighborhoods provided 5,159 co-occurrences involving 774 taxa. Transposase pairs with a conserved genomic context also have a higher average amino acid sequence identity (95.2 ± 5.5% versus 89.5 ± 6.3% for pairs in differing locations), further supporting our premise that these co-occurrences reflect recent divergence within a vertical lineage. Therefore, only those pairs within different gene neighborhoods were included in the microbial social network and analyzed further. This strategy minimizes, but cannot completely rule out, the possibility of vertical inheritance in closely related taxa. The observed non-conservation of the surrounding regions could have resulted from various combinations of events, including transposase relocation and/or loss in either or both species. Furthermore, we tested and confirmed the efficiency of the neighborhood approach in minimizing the effects of vertical inheritance by collapsing strains belonging to the same genus and habitat (see Materials and methods).

**Figure 3:**
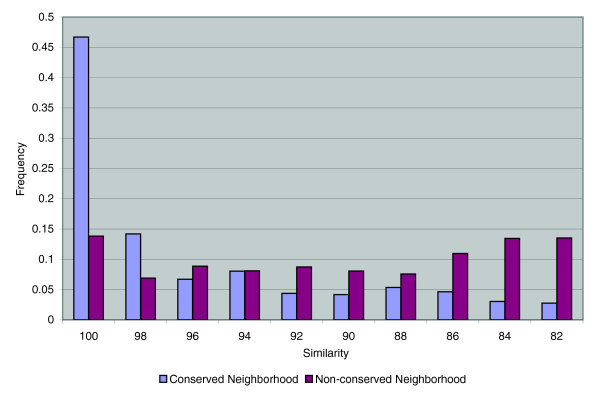
Comparison of transposase connection protein identity for pairs in conserved neighborhoods (blue) versus non-conserved neighborhoods (red). The sharp drop-off in identity for pairs in conserved neighborhoods suggests a rapid loss of transposases, while the lower identity for pairs in non-conserved neighborhoods suggests an acquisition of transposases from a diverse population of transposases.

The distributions of sequence identities of shared transposases in conserved and non-conserved neighborhoods are also strikingly different (Figure 3). There is a sharp drop in sequence identity for the conserved neighborhood set from the 98-100% category to the remaining categories. This short half-life of (most probably) clonal transposases suggests that there is little or no selection for these sequences in the genome. For the shared transposases in non-conserved neighborhoods, high-identity transposases are less common. This is generally consistent with the premise that these shared transposases are not clonal; that is, they are drawn from a population of transposases with a certain degree of sequence variation.

### Transposase identity versus phylogenetic distance

For genes transmitted by vertical inheritance, sequence identity decreases with increasing phylogenetic distance between organisms. Since genes acquired through lateral transfer do not follow this pattern, we investigated how the level of sequence identity of our selected shared transposases correlates with the phylogenic distance between their source taxa. In the case of multiple shared transposases, we considered the highest identity.

We used two measures of phylogenetic distance; one based on 16S RNA calculated using PHYLIP [15], and one based on the average amino acid identity (AAI) of a set of 31 marker genes used for tree reconstruction [16]. The average protein identity is more sensitive than 16S identity when comparing closely related taxa [17]. The latter method is suitable for this study since the metrics are directly comparable to transposase identity, and since there are many closely related taxa.

Using the first measure, sequence identity is observed to decrease with increasing phylogenic distance for low to medium phylogenetic distances (<40). The correlation coefficient is weak, but negative (-0.067), and the average transposase identity is 89.5 ± 6.3%. For large phylogenetic distances, such as that between the Bacteria and the Archaea (>80) the average transposase identity is 89.7 ± 7.8%. Thus, the negative correlation coefficient reflects the presence of many very closely related taxa that share transposases with high sequence identity, rather than a tendency for distant taxa to have dissimilar transposases. As a control, we also studied the correlation between phylogenetic distance and transposase similarity for the transposases in conserved neighborhoods and found a stronger correlation at -0.11. This supports the notion that the transposases that are not in conserved neighborhoods are not primarily results of vertical inheritance. This decoupling of phylogenic distance from sequence identity again suggests that some, if not most, of the shared transposases that were not found in conserved neighborhoods were acquired by lateral transfer.

**Figure 4:**
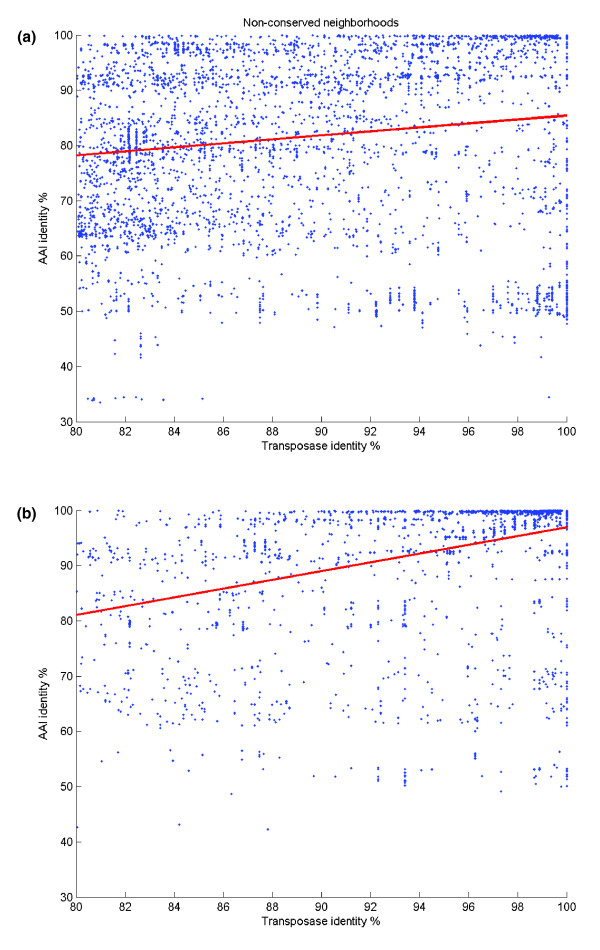
Comparison of average amino acid identity of 31 marker genes versus transposase amino acid identity for connections in **(a)** non-conserved and **(b)** conserved neighborhoods. The correlation coefficient for (a) is 0.145 and 0.385 in (b), suggesting that connections in non-conserved neighborhoods are less likely to be due to vertical inheritance.

Using the second measure, we find an even clearer case of a stronger correlation between transposases in conserved neighborhoods and phylogenic distance. The correlation coefficient in non-conserved neighborhoods was 0.145 versus 0.385 in conserved neighborhoods (Figure 4). Additionally, the average AAI between taxa with shared transposases in conserved neighborhoods is higher at 93%, significantly (*t*-test, *P *< 10^-3^) higher than 82% in the non-conserved set. This again suggests that by discarding connections where transposases are in conserved neighborhoods, we reduce the effect of vertical transfer of transposases.

Since the 31 marker genes are most likely not a result of lateral transfer, we can compare the average identity of our transposases to the distribution of total AAI to indicate the degree of transfer into the unconserved neighborhoods. The average transposase identity in unconserved neighborhoods is significantly (*t*-test, *P *< 10^-3^) higher than the AAI of the marker genes (81.6 ± 15.7%), suggesting that it is reasonable to assume that the majority of transposases are results of lateral transfer and not vertical inheritance.

### Shared transposases within shared habitats

Most of the taxa included in this study are associated with a habitat. The four most common habitats in the Genomes OnLine Database (GOLD) [18] are host, marine, soil, and aquatic; combined they constitute 1,302 of the 1,858 habitat assignments in the full Integrated Microbial Genomes (IMG) database [19]. Most of the other habitats can be classified as subtypes of these four super-habitats (Table S2 in Additional data file 1). For instance, bacteria categorized as intestinal flora would also fall within the host super-habitat. Microbes found to be viable in multiple habitats can be assigned to more than one super-habitat.

Most taxa that share a transposase are found to also share a habitat (Table S3a in Additional data file 1). In Figure 2 for instance, item 1 is an example of an intra-habitat connection, where *E. coli *strains within the same habitat share transposases. Specifically, 41% of all transposase connections occur between organisms with identical habitat assignments, significantly (*P *< 10^-3^) more than the 22% expected if we were to randomly pick connections from the network. Likewise, partially overlapping habitats account for 25% of the total, also significantly (*P *< 10^-3^) more than the 19% expected from random processes. Over-representation of both of these groups suggests, perhaps not surprisingly, that a shared habitat facilitates lateral transfer of transposases between microbes, and that microbes found in more than one habitat have the opportunity to exchange with microbes in each of those habitats. These patterns persist also when the transposase identity cutoff is increased to 90% (Table S3b in Additional data file 1).

While the picture is not completely clear, the transposase co-occurrence data suggest that taxa assigned to the host habitat transfer transposase genes more often than do taxa assigned to other habitats (1,552 connections versus the expected 921, *P *< 10^-3^). Several factors likely contribute to this. First, for many pathogens and symbionts, the host habitat is not their only habitat. They can also live outside their hosts in a secondary environment where they have the opportunity to interact with the members of a different microbial community. For instance, green algae in the Great Lakes have been found to harbor several enterobacterial pathogens [20] that may at some point again return to the host environment. This alternation between host and external environment could have occurred repeatedly, thus providing repeated opportunities for transposase transfer to/from different organisms within these bacterial lineages. Second, some bacteria have been shown to regulate the rate of transposition in response to stress, increasing the frequency when the genomic alterations resulting from transposition may prove advantageous [21]. Thus, it may be that pathogens, with their perennial need to adapt to host defenses and antibiotics, employ more frequent IS and transposase exchange.

### Cross-habitat connections

Of particular interest, 35% of the transposase co-occurrences are found in taxa that are not known to share any habitats. These connections that bridge between different habitats are discussed in the following sections. In some cases, the specific transposases are reported. For other cases, an extensive list of transposase connections is provided in Additional data file 2. In Figure 2, item 2 signifies a cross-habitat connection between *Bacillus cereus *(soil) and *E. coli *(host), and *Bacillus thuringiensis *(host, soil) with *B. cereus *(soil).

#### Soil-aquatic connections

We observed 27 instances where transposase connections link a microbe annotated as soil with one annotated as aquatic. In at least some cases, these observations suggest that the current annotations are incomplete. For example, despite their differing annotations, *Acidovorax *(soil) and *Bordetella *(aquatic) strains have been observed together in arctic soil [22]. We also found connections (specifically IS4 transposases) between *Bordetella *(aquatic) and *Pseudomonas *(typically soil and/or host) strains with non-overlapping annotated habitats, but both *Bordetella *and *Pseudomonas *strains have been observed in community shifts within a bioreactor processing industrial wastewater [23]. We note that the aquatic and soil habitats have many opportunities to overlap as rainwater moves through land on its way to the oceans (for example, in agricultural runoff, floodplains).

#### Host-soil connections

There are 414 co-occurrences connecting microbes assigned to host with those assigned to soil. Of these, 70 connections are between *Burkholderia *species and thus may simply be cases of vertical transmission followed by rearrangements and/or deletions. Those species annotated as host include *Burkholderia enocepacia, Burkholderia cepacia*, and *Burkholderia mallei*, those as soil are primarily *Burkholderia pseudomallei *strains. Although *B. pseudomallei *is observed in rice paddies [24], we suggest that it should also be classified as host since it has been linked to diseases such as melioidosis in humans. Further support for such a host habitat assignment comes from its observed connections to other pathogens outside the *Burkholderia *family (for example, *Shigella dysenteriae*, via ISSfl2, ISSfl4 and other transposases), connections that are more difficult to dismiss as vertical inheritance. We also see transposases shared between more distant taxa that are not known pathogens. *E. coli *strains share transposases (COG3547) with *Burkholderia thailandensis *and *Burkholderia vietnamiensis*, *Ralstonia solanacearum*, and the even more distant *B. cereus*. With the exception of strain O157:H7, the *E. coli *strains are non-pathogenic; the other four also are not known pathogens, although some of their close relatives are [25-27]. While it is unlikely that these microbes come into regular contact with each other within hosts, it is plausible that they may interact outside of hosts, for instance in agricultural manure [28] or waste water.

#### Host-aquatic connections

We find 97 instances where transposases are shared between organisms assigned to the host and aquatic habitats. Just as the host-soil connections discussed above are dominated by *Burkholderia *species, host-aquatic connections are dominated by 6 *Vibrio cholera *strains annotated as aquatic. These strains connect with *E. coli *strains (IS5 and others), the *Wolbachia *endosymbiont (IS4), and the Firmicute *Staphylococcus aureus *(IS5). Three of them connect to *Providencia stuartii *(IS605), suggesting a recent transfer, either when or shortly after those *V. cholera *strains diverged. Although *V. cholera *is annotated as aquatic, none of the strains were found to share transposases with any other aquatic species. Since it is a known pathogen ([29] and references therein) and the causative agent of cholera, infection of a human host could provide the opportunity to interact with host species such as *E. coli*. The connection to *Wolbachia *is especially interesting, as *Drosophila *is a host for *V. cholera *infection [30] as well as for the *Wolbachia *endosymbiont. Hence, as with *B. pseudomallei *previously, our observations indicate that *V. cholera *should be assigned to the host habitat, as well as to aquatic.

#### Host-marine connections

Transposases are shared between host and marine species in 159 cases. Since fecal contamination of marine environments is known to occur [31,32], it is not surprising to see connections involving host organisms such as *E. coli, Shigella dysenteriae*, and *Yersinia pestis*. However, the host organisms also include symbionts that reside in plant root nodules, such as *Rhizobium *and *Mesorhizobium *species. Interestingly, the soil species *Sinorhizobium medicae *and *Sinorhizobium meliloti *also connect to marine species, although not to the same targets as *Rhizobium *and *Mesorhizobium*.

#### Soil-marine connections

There are 48 co-occurrences between soil and marine organisms. The pattern here is similar to that observed for host-marine connections in that they both involve organisms related to soil and agriculture. The organisms annotated as soil include the nitrogen-fixing *Sinorhizobium *and *Bradyrhizobium*, both of which are found in plant root nodules, thus suggesting that they, like *Rhizobium *and *Mesorhizobium*, should be assigned to host. Also annotated as soil is *Paracoccus denitrificans*, a nitrogen-oxidizing bacterium often found in soil sludge.

#### Aquatic-marine connections

The majority of the 152 connections between marine and aquatic organisms are found to occur between aquatic *V. cholera *and marine *Shewanella *or between the *Photobacterium *and *Vibrio *genera within the Vibrionaceae family. It is difficult to draw a clear line of demarcation between these two, often contiguous habitats that are distinguished by their salinity, temperature and nutrients. *V. cholera *strains, for instance, can be found in coastal regions [33] that form an indistinct interface between aquatic and marine. This situation may be reflected in the number of closely related groups whose annotations span the aquatic and marine environments, such as the Vibrionaceae, the *Cyanothece*, and the Rhodobacteraceae (Roseobacter and Dinoroseobacter).

#### Generalists

Organisms annotated as viable in several habitats are likely to be versatile enough to adapt to changing conditions as well as diverse environments, thus having the opportunity to interact with more different microbial communities. To test this supposition, we analyzed the transposase connections of all the organisms in our network that are annotated as aquatic-host-soil. These organisms belong to only ten genera: *Chromobacterium*, *Citrobacter*, *Clostridium*, *Klebsiella*, *Mycobacterium*, *Novosphingobium*, *Pseudomonas*, *Ralstonia*, *Salmonella*, and *Yersinia*. Collectively, they have 908 connections to organisms outside this group. Of these 908, 772 involve enterobacterial species (*Klebsiella*, *Salmonella*, *Yersinia*) and, with few exceptions, connect to host organisms. Thus, despite their generalist annotation, most of them are not found to interact often with organisms outside the host habitat. There are exceptions, including the pathogen *Ralstonia pickettii *with diverse connections that range from the soil bacterium *Arthrobacter aurescens *to the host and soil Burkholderia species and even to a eukaryote pathogen, *Plasmodium yoelii*. Similarly, *Pseudomonas aeruginosa *shares transposases with a variety of soil and host bacteria, and also one marine bacterium (*Photobacterium *sp. SKA34). Nevertheless, it appears that a generalist annotation does not, in itself, imply a wide range of interactions.

### Cross-phylum connections

Transposases tend to be constrained to specific phylogenetic groups (Table S4a,b in Additional data file 1). Indeed, 91% of the shared transposases observed in this study are shared between members of the same phylum. This may reflect a lower frequency of cross-phylum transfers due to the obstacles posed by increasingly divergent genome arrangements, DNA polymerases, or genomic nucleotide bias. Furthermore, since we included only the reciprocal best hits with at least 80% identity, it is likely that we have selected for more recent transfers. The ISFinder resource [34,35] contains detailed information about the distribution of various IS families, including those with lower levels of identity. It is likely that this resource will reveal transposase connections that are below our detection levels, extending the network. In Figure 2, we observe a cross-phylum connection at item 3 between *B. cereus *and *E. coli*, and also to *P. aeruginosa*.

In our study of high-identity transposases, we observe 448 co-occurrences between members of different phyla. Of these, 441 involve connections linking Proteobacteria with Firmicutes, Cyanobacteria, Actinobacteria, or the eukaryote Apicomplexa. This prevalence of proteobacterial connections is not surprising since the sequenced genomes available are heavily biased toward that group.

There is a tendency for these connecting taxa to share the same or similar environments. For example, the actinobacterium *Corynebacterium diphtheriae*, annotated as host, connects to 15 Proteobacteria, 14 of which are found in host or host-related environments. Likewise, cross-connections between Firmicutes and Proteobacteria are seen mostly between host species. For instance, Proteobacteria link to the following Firmicutes, all of which are annotated as host: *Clostridium bolteae *ATCC BAA-613, *Clostridium ramosum *DSM 1402, *Clostridium scindens *ATCC 35704, *Staphylococcus aureus *subsp. *aureus *NCTC 8325, and *Streptococcus *strains. An intriguing exception involves the soil Firmicute *B. cereus *ATCC 10987, which not only connects to enterobacterial species, most of which are annotated as host, but also to *Beggiatoa *sp. and *Desulfotalea psychrophila*, both of which are annotated as marine. It is not clear, however, if any environment promotes cross-phylum connections more than any other, due to the bias towards sequencing organisms from certain environments such as host.

One example of a cross-phylum connection not involving a Proteobacterium is provided by *Fusobacterium nucleatum*, which connects to the Firmicutes *Clostridium perfringens *and *Staphylococcus haemolyticus*. All three share the host habitat, and *C. perfringens *is also noted as soil. Other examples can be found within the Cyanobacteria, where *Synechococcus *sp. WH 7805 (marine) connects to several *Shewanella *species (marine) and to *Vibrio *species (aquatic).

### Bridging connections

In characterizing our microbial social network, we also identified those taxa that do not share a transposase but that are connected via a third 'bridge' taxon. For example, suppose that taxa A and C have no transposases in common, but A shares a transposase with B and C shares a different transposase with B. Taxon B is thus the bridge connecting taxa A and C. From this we infer that B has shared a habitat with A at some time in the past, and likewise with C. Furthermore, as a result of this bridge, there exists a possibility of gene flow between A and C via B. Item 4 in Figure 2 shows *E. coli *B171 as a bridge between *B. cereus *ATCC 10987 and *P. aeruginosa *2192.

We tallied the number of two-paths for which each taxon serves as the bridge between two taxa that are not directly connected. The 30 species with the highest scores are ranked (Table S5 in Additional data file 1). The top three are the soil bacterium *B. cereus*, followed by *Streptococcus pneumoniae *SP19-BS75 (host) and *B. vietnamiensis *G4 (soil). The first marine and aquatic species are ranked 11th and 12th, respectively. There is no apparent correlation between the number of bridging connections and the number of habitat annotations a species may have. The highest ranking generalist (annotated as soil, aquatic and host) is *P. aeruginosa *PA7 at position 22.

The microbes that have the greatest impact on the social network are not necessarily those with the highest number of connections, either direct or bridging. Bacteria such as *Shigella*, *Escherichia*, and *Salmonella *have many connections, but most of them are within the Enterobacteriaceae and thus do little to expand the network. More significant are those that bridge between different groups or families, such as *Streptococcus *and *Staphylococcus *that bridge between the Firmicutes and the large clusters of Proteobacteria within the host habitat.

## Discussion

Some instances of the multiplicity of microbial interactions, either between or within habitats, have been observed directly, but, to the best of our knowledge, our network of shared transposases is the first large-scale attempt to use genomic records to infer genetic interactions shared between organisms. By identifying pairs of taxa that share a transposase that was likely acquired by LGT, we generated a microbial interaction network including almost 800 organisms. The results suggest a tendency to transmit transposases and their associated ISs most frequently to other organisms within the same habitat.

The primary source of potential error in our analyses stems from including transposase pairs that were acquired by vertical inheritance, most likely in closely related taxa. However, this source of error was reduced by excluding transposase pairs that resided in conserved genomic neighborhoods. Furthermore, the analysis identified many connections between distantly related taxa that have no recent common ancestor. The results are inevitably biased due to the limited number of sequenced microbial genomes and their skewed selection. Since we are far from having genome sequences representative of the complete tree of life, it is likely that many interesting organisms and habitat types are missing from this study. Some of these organisms may serve as vectors of transmission between those studied in this work. With an increasing rate of sequencing, many of these gaps will no doubt be resolved.

In addition to the connections between taxa with shared habitats, we also observe connections between taxa found in physically different environments. These observations are consistent with findings from microbial ecology and suggest that there is a degree of mixing of microbes between environments, although probably not to the extent that 'everything is everywhere' [36], since transposases are most often shared within their environment. In some cases, this microbial relocation can be attributed to well-documented mechanisms (for example, the lifecycle of *V. cholera*, the movement of agricultural drainage water). Other, less obvious instances merit further investigation.

These data suggest that a number of microbes annotated as host should be re-annotated to reflect their cyclical movement between, and adaptation to, a host and an external environment. For that purpose we suggest a new category: host-external cycle - for example, host-soil cycle for pathogens who spend parts of their natural life cycle in a soil environment. Numerous examples of this can be found among the host-soil cross-connections, including the enterobacterial pathogens that cycle between animal intestines and soil. Likewise, *V. cholera *exemplifies this cycling between host and marine environments. Adoption of this new habitat category would not only acknowledge that many pathogens periodically change environments, but would also distinguish between obligate and non-obligate pathogens and parasites. (Since very few shared transposase connections were found among obligate pathogens, this group is largely absent from the network observed in this study.)

Distantly related taxa with shared transposases tend to have similar habitat annotations. Notably, all connections over very great phylogenetic distances, such as between the Proteobacteria and the Apicomplexa, involve pathogens or parasites. This suggests that frequent and/or intimate co-habitation may be necessary to facilitate transmissions that are less likely given the large differences in the genomic structure, nucleotide bias, regulatory mechanisms, DNA polymerases, and so on, between the two organisms.

## Conclusions

This analysis of the transposase LGT history recorded in microbial genomes expands our vision of the microbial world in several ways. First, we see that current habitat annotations can be too restrictive and thus fail to represent the full extent of a microbe's habitat, and we also suggest refinements to the pathogens in particular. Second, we see that microbes who naturally traverse different habitats, such as many of the pathogens, also share transposases with microbes from the various environments. Therefore, it is likely that they may also import other DNA, including protein-coding genes, from its secondary environment. Third, it suggests that the impact of LGT could be more far-reaching than previously thought, since gene acquisitions are not limited to the immediate vicinity, but can be drawn from different environments. Fourth, this is a tentative survey into a molecular basis for microbial ecology, an area that has not received much attention so far and that hopefully will expand in the future with more sequenced genomes and metagenomes.

## Materials and methods

### Identifying transposase co-occurrences

The IMG database [19] is the most comprehensive database of microbial genomes, with over 800 finished and draft genomes as of this writing. For this study, we used BLAST [37] to compare all genomes against each other and to identify cases where both BLAST reciprocal best amino acid hits belonged to one of the 26 clusters of orthologous genes (COGs) [38] annotated as transposases. The use of reciprocal best hits ensures that we select the most similar transposases between each pair of taxa, which is useful given the mobility of these sequences. The threshold for sequence identity was set at 80%. Frequently, there are more than one transposase connecting two taxa. In this case, we retain the transposase with the highest identity, which would suggest the most recent transposition. Furthermore, to avoid selecting fragments or short, local alignments, we set a bit score [37] cutoff at 80. This corresponds to an e-value of <10^-20 ^given a database size of over 4 million sequences in IMG. An extensive list of all transposase connections, along with details of individual transposase connections and their e-values are provided in Additional data file 2.

Of particular interest are connections that bridge between distant taxa or distinct habitats. For example, suppose that transposase T_1 _occurs frequently in bacterial group A, whereas transposase T_2 _is found in a distant bacterial group, group B. If one organism in group B also contains transposase T_1_, it could serve as a bridge to indirectly connect the other members of group B with group A. In graph theory, this is known as a two-path, where two nodes (in this case, two taxa) are not connected directly, but only via a third node (taxon). Two-paths are easily found using adjacency matrices. In the adjacency matrix A, A_ij _= 1 when there is a direct connection between nodes *n*_*i *_and *n*_*j*_. If we set the second Markov transition step X = AA', then X_ij _= 0 and A_ij _> 0 when *n*_*i *_and *n*_*j *_are connected exclusively by a two-path.

### Interaction graphs

The complex network of transposase co-occurrences can be represented as a graph of nodes and edges, where taxa are represented as nodes and shared transposases are represented as connecting edges. From the BLAST reciprocal best hits, we created a co-occurrence graph with *N *= 774 nodes and *E *= 5,159 edges, as well as analogous graphs for subsets selected by habitat. To compare the appearance of transposase networks with networks of genes that are presumed to be much less mobile, we created networks of genes associated with transcription (category K) and three COGs from other less conserved functions (categories P, E and R, associated with transport and general predictions). We found that genes coding for essential functions, such as polymerases, formed very compact networks, where almost every taxa is connected to each other. Conversely, less conserved genes (in this case ion transport) rarely find connections outside their host at a threshold of 80% identity. If they do, it is very often to a closely related organism. The tranposase network can span large phylogenetic distances, although miss connections with much more closely related organisms, setting it apart from networks of polymerases and less conserved genes.

Graph data in the *Medusa *[39] format are provided as Additional data file 3 for visualization of the transposase network. The network is also provided in raw text format as Additional data file 4. Information and references for all isolate genomes used in this study can be found at IMG [40].

### Gene neighborhoods and phylogenetic distance

Gene neighborhoods were defined as a stretch of protein coding sequences with intergenic distances less than or equal to 300 bp. When two transposases in two taxa were found in the equivalent neighborhoods, we assumed a high likelihood of vertical inheritance, and the transposase connection was removed from this study. Details of the methodology used to determine gene neighborhoods are available at the IMG website [40].

We used the program DNADIST in the PHYLIP package using the Kimura substitution model on the 16S RNA sequence alignment. The alignment is the subset of sequences from the database SILVA [41] corresponding to the genomes in our study. The AAI for the 31 marker genes [16] were calculated analogously to the transposase identity above.

### Effects of closely related taxa

Since the corpus of sequenced genomes is biased towards certain genuses, it is conceivable that this bias is also reflected in the transposase connection study. For instance, we have multiple strains of *E. coli*, *S. enterica*, *S. aureus *and *S. pneumoniae *and several others. When several members of a genus all share the same habitat, then the presence of inherited transposases may skew the habitat cross-connection towards same-habitat connections. Although the problem of inherited transposases has been addressed by removing connections between transposases in conserved neighborhoods, an additional control is to remove superfluous closely related organisms from the network and study the habitat connections. For this purpose, we collapsed genomes within the same genus and habitat (for instance *E. coli *K12 and O157:H7) into a representative node and recalculated the connection scores. We found that the patterns of connections (Table S6 in Additional data file 1) were largely consistent with the case where genomes are not collapsed by genus (Table S3a in Additional data file 1). As before, within-habitats are overrepresented (observed, 552; expected, 329; *P *< 10^-3^). This suggests that transposases more often spread within a habitat than between habitats regardless of sequencing biases, and also supports the removal of connections between transposases in conserved neighborhoods as an efficient method of reducing effects of vertical transposase inheritance.

## Abbreviations

AAI: average amino acid identity; COG: cluster of orthologous gene; GOLD: Genomes OnLine Database; IMG: Integrated Microbial Genomes database; IS: insertion sequence; LGT: lateral gene transfer.

## Authors' contributions

SDH designed the project and performed the analysis. KM contributed to the computational analysis. NCK contributed to the biological analysis.

## Additional data files

The following additional data are available with the online version of this paper: Tables S1-S6 (Additional data file 1); an extensive list of the specific transposases present in various taxa (Additional data file 2); the raw network data in Medusa format (Additional data file 3); the raw network data in text format (Additional data file 4).

## Supplementary Material

Additional data file 1 Tables S1-S6. Click here for file

Additional data file 2 Specific transposases present in various taxa. Click here for file

Additional data file 3 Raw network data in Medusa format. Click here for file

Additional data file 4 Raw network data in text format. Click here for file
